# Heterogeneous trajectories of intrinsic capacity and their predictors: a Bayesian network analysis of a longitudinal cohort in Chinese nursing homes

**DOI:** 10.3389/fpubh.2026.1755509

**Published:** 2026-03-25

**Authors:** Hong Su, Yuefeng Wang, Yuke Zhang, Xiaona Qi

**Affiliations:** 1 School of Nursing, Daqing Campus, University of Harbin Medical, Daqing, China; 2 Department of Nursing, The Cancer Hospital of Harbin Medical University, Harbin, China

**Keywords:** aged, Bayesian network model, developmental trajectory, intrinsic capacity, nursing homes

## Abstract

**Objectives:**

To identify distinct developmental trajectories of intrinsic capacity (IC) among nursing home residents in China and to elucidate the complex network relationships among their predictors using a Bayesian network model, guided by the Socio-Ecological Theory.

**Methods:**

An 18-month longitudinal study was conducted with 592 older adults from three nursing homes in Northeast China from March 2022 to February 2024. IC was assessed at baseline (T0), 6 (T1), 12 (T2), and 18 (T3) months using the ICOPE screening tool. Latent growth mixture modeling (GMM) identified trajectory subgroups. A BN was constructed to model the interrelationships among significant predictors identified from multinomial logistic regression.

**Results:**

Three distinct IC trajectories were revealed: ‘High Functioning-Stable’ (16.0%), ‘Medium Functioning-Slow Decline’ (40.5%), and ‘Low Functioning-Rapid Decline’ (43.4%). The Bayesian network model identified four direct predictors of trajectory membership: family function, number of chronic diseases, grip strength, and perceived stress. Health-promoting behaviors and loneliness were key indirect predictors, operating through mediators such as grip strength and perceived stress, respectively.

**Conclusion:**

Intrinsic capacity development among nursing home residents is heterogeneous and driven by a complex network of interlinked factors. Our findings advocate for a paradigm shift from single-factor interventions to integrated, multi-target strategies. Clinicians should simultaneously address family support, multimorbidity, physical capacity, and psychological well-being to effectively delay IC decline.

## 1 Introduction

China has entered a stage of moderate population aging. According to the National Bureau of Statistics, by the end of 2023, approximately 290 million people were aged 60 and above, accounting for 21.1% of the total population, while the population aged 65 and above reached 210 million, representing 15.4% (1). Evolving family structures and the health pursuits of older adults are driving a shift in care models from a traditional family-based system to one involving multiple social entities, including residential institutions and community-supported care (2). A significant proportion of older adults experience adverse outcomes following relocation to nursing homes. Studies indicate that 25.6% face mortality, often attributable to factors such as advanced age, poor health, physical and cognitive impairments, depression, and low participation in activities (3, 4). Furthermore, 38.2% of mortality cases have been associated with a decline in intrinsic capacity (5). Therefore, enhancing intrinsic capacity, reducing mortality, and improving the quality of life for older adults residents in care institutions is an urgent requirement and a critical imperative for fostering a healthy aging society.

Intrinsic Capacity (IC), a concept central to the World Health Organization’s (WHO) framework on healthy aging, refers to the composite of all an individual’s physical and mental attributes that can be mobilized at any given time (6). It is constituted by five domains: locomotion, cognition, psychology, vitality, and sensory capacity. IC and its individual domains reflect the overall health status and dynamic health trajectories of older adults. Substantial evidence links a higher level of intrinsic capacity to a reduced risk of disability and an improved overall quality of life. Specifically, for every one-unit decline in intrinsic capacity, hospitalization rates increase by 1% and mortality rises by 2% among the older adults (7, 8). Intrinsic capacity is generally low among older adults in care institutions, a phenomenon primarily driven by admissions motivated by difficulties in home care, personal functional decline, or multimorbidity. Thus, this group inherently represents a high-risk population with vulnerable intrinsic capacity. Maintaining a high level of intrinsic capacity is crucial for institutionalized older adults to sustain survival (i.e., reducing mortality) and preserve functional autonomy (i.e., delaying disability). A one-year follow-up study of 800 older adults in Southern France by Juan et al. revealed that a decline in intrinsic capacity was significantly associated with mortality among nursing home residents, whereas maintaining higher vitality capacity could mitigate this risk (5). As a dynamic construct, longitudinal monitoring of the developmental trajectory of intrinsic capacity and its related factors in this population is crucial for developing personalized interventions and implementing comprehensive management strategies to mitigate adverse outcomes like hospitalization and mortality in nursing homes.

The development of intrinsic capacity results from the complex interplay between individual characteristics and environmental factors. Existing research has identified factors such as age, nutritional status, educational attainment, physical activity levels, handgrip strength, history of falls, activities of daily living, and multimorbidity as significant determinants influencing the trajectory of intrinsic capacity (9, 10). In contrast, social factors, including elements such as family functioning and social networks, and their role in enhancing self-worth and dignity, promoting healthy behaviors, and improving the physical and mental health of older adults in nursing homes, have received comparatively less attention. Several qualitative studies have identified that the high perceived stress experienced by the older adults—stemming from loneliness and multiple losses (e.g., loss of health, privacy, autonomy, and their original social circles)—is a significant factor affecting their overall well-being (11); however, these findings have not yet been quantitatively validated. Furthermore, the application of theoretical frameworks to systematically explore the determinants of intrinsic capacity trajectories among nursing home residents remains markedly limited in the existing literature. Consequently, the adoption of a clear and comprehensive theoretical framework is imperative to identify the principal determinants of intrinsic capacity development in this population, constituting a critical step for formulating targeted, evidence-based interventions.

The Socio-Ecological Theory (SET) provides a robust theoretical framework for exploring the interactions between individuals and their environments from a holistic and systemic perspective (12). The SET conceptualizes a concentric nested structural model centered on individual factors to explain how person-environment interactions shape individual behaviors and outcomes. This model stratifies the ecological environment into three primary systems: the microsystem (e.g., age, educational level, perceived stress, number of chronic conditions, grip strength, loneliness, and health-promoting behaviors), the mesosystem (e.g., marital status, family functioning, social networks), and the macrosystem (e.g., health insurance and pension insurance) (13). The SET model has been effectively utilized to analyze overall well-being in older adults in certain contexts. However, it has not yet been applied to quantitatively investigate the determinants of intrinsic capacity trajectories among nursing home residents. Furthermore, how these determinants interact and their combined effects on distinct subgroups within these trajectories remain unclear. Guided by the SET framework, this study incorporates multi-level determinants and employs a longitudinal design to construct a Bayesian network model. It aims to (1) analyze factors influencing the developmental trajectories of intrinsic capacity among nursing home residents from individual, familial, and societal perspectives; (2) examine the network relationships among these factors; and (3) identify key modifiable targets to provide a scientific basis for developing interventions to maintain intrinsic capacity, delay the onset and progression of disability, and prevent adverse outcomes such as falls, hospitalization, and mortality.

## 2 Methods

### 2.1 Aims

Guided by the Socio-Ecological Theory, this study aims to analyze the developmental trajectories of intrinsic capacity among nursing home residents and examine the network relationships among factors influencing distinct trajectory subgroups.

### 2.2 Design

A longitudinal study design was used.

### 2.3 Participants

From March 2022 to February 2024, participants were recruited by convenience sampling from three nursing homes in Heilongjiang Province, northeast China. While convenience sampling has inherent limitations regarding generalizability, it was deemed the most practical and feasible approach for this initial longitudinal study within institutional settings, given the challenges associated with random sampling in nursing homes (e.g., access constraints, the need for sustained participation over 18 months). Inclusion criteria: (1) age ≥ 60 years; (2) length of residence in the nursing home≥ 3 months; (3) voluntary participation. Exclusion criteria: Individuals who were medically diagnosed with psychiatric conditions or had difficulty in communicating and severe cognitive impairment, as indicated by a Mini-Mental State Examination score <9, were excluded.

Sample size was calculated using the random sample size formula: *n* = u^2^_α/2_*π*(1—π)/δ^2^ (14), *n*: estimated sample size; π: population rate, according to the literature review (15), the average of the lower limit estimate (67.9%) and the upper limit estimate (77.4%) of the prevalence of intrinsic capacity decline in the older adults population in our country is considered, which is 72.65%; u_*α*/2_ is the value at the significance level *α* = 0.05, where *u*_α/2_ = 1.96; *δ* represents the allowable error, which is set at 5%. To account for a 20% loss to follow-up and invalid questionnaires, the baseline sample size was calculated to be no less than 527 participants.

### 2.4 Data collection

Data were collected through face-to-face interviews. After the researchers obtained informed consent from the participants, the author conducted the questionnaire survey with the support and safety ensured by a care staff, requiring approximately 30–40 min. During the survey process, the researcher helped in the completion of the questionnaire for participants with reading difficulties by reading each question aloud and offering them a standardized explanation if necessary. The researchers clarified all points and concerns raised by the participants. In accordance with the ICOPE recommendation for semi-annual assessments of intrinsic capacity in older adults, follow-up surveys were conducted every 6 months, resulting in three follow-up sessions. The baseline assessment (T0) took place in March 2022, followed by subsequent assessments at 6 months (T1), 12 months (T2), and 18 months (T3) post-baseline. All questionnaires are collected on the spot and checked for completion.

### 2.5 Measurement

#### 2.5.1 General information questionnaire

The general information questionnaire was a self-designed questionnaire that was developed by considering the theoretical framework of the study and a review of relevant literature. The questionnaire included variables such as age, gender, average monthly income [categorized based on the local average pension level: low (<1,000 rmb), average (1,000–3,000 rmb), high (>3,000 rmb)], education level, marital status, number of chronic diseases, self-rated health status, smoking habits, alcohol consumption, sleep quality, medical insurance and pension insurance which was further refined based on feedback obtained from 15 participants in a preliminary survey to ensure comprehensibility.

#### 2.5.2 The University of California at Los Angels Loneliness Scale (UCLA Loneliness Scale)

The UCLA Loneliness Scale used to assess loneliness was developed by Russell in 1980 and later revised by Wang (16). The questionnaire consists of 20 items and were scored from 1 (never) to 4 (always), with higher scores indicating greater levels of loneliness. The total score ranges from 20 to 80 and can be categorized as follows: 20–34 indicates low loneliness, 35–49 indicates moderate loneliness, and 50–80 indicates high loneliness. The scale has demonstrated strong reliability, with a Cronbach’s alpha coefficient of 0.92 (17). In the current study, the internal consistency reliability (Cronbach’s alpha) was 0.82.

#### 2.5.3 Chinese perceived stress scale (CPSS)

The CPSS was used to assess the perceived stress, which was developed by Cohen in 1983 and revised by Yang and Huang (18). The scale consists of 14 items and measures two dimensions: tension and a sense of being out of control. It employs a 5-point Likert scoring system, with total scores ranging from 14 to 70. Scores can be categorized as follows: 14–26 indicates low perceived stress, 27–41 indicates moderate perceived stress, and 42–70 indicates high perceived stress. In this study, the scale demonstrated good reliability, with a Cronbach’s alpha coefficient of 0.86.

#### 2.5.4 Lubben social net-work scale-6 (LSNS-6)

The LSNS was developed by Lubben et al. (19) and translated into Chinese in 2018 (20). The scale comprises 6 items, each rated on a 6-point Likert scale ranging from 0 to 5. The total score ranges from 0 to 30, with scores below 12 indicating social isolation among older adults. In this study, the scale demonstrated good internal consistency, with a Cronbach’s alpha coefficient of 0.83.

#### 2.5.5 Family APGAR index (ARGAR)

ARGAR, which was developed by Smilkstein (21). This questionnaire consisted of five dimensions: adaptability, cooperation, growth, emotionality, and closeness. Each item is rated on a 3-point scale ranging from 0 to 2. The total score ranges from 0 to 10, with the following classifications: 0–3 indicates severe family dysfunction, 4–6 indicates moderate dysfunction, and 7–10 indicates good family functioning (22). In this study, the scale demonstrated good internal consistency, with a Cronbach’s alpha coefficient of 0.81.

#### 2.5.6 Health-promoting lifestyle profile II (HPLP-II)

The HPLP-II was developed by Walker et al. (23) and translated into Chinese by Cao et al. (24). It consists of 40 items categorized into six dimensions: physical activity, self-realization, nutrition intake, interpersonal support, health responsibility, and stress management. Each item is rated on a 4-point Likert scale, with total scores ranging from 40 to 160. A total score of ≥100 indicates healthy behavior, while a score of <100 indicates unhealthy behavior. In this study, the scale demonstrated good internal consistency, with a Cronbach’s alpha coefficient of 0.87.

#### 2.5.7 Integrated care for older people (ICOPE) screening tool

The ICOPE screening tool was used to assess the Intrinsic Capacity of residents in nursing home (25). It is composed of five dimensions (1) Cognition: two items about orientation and memory were spared from Mini-Mental State Examination (MMSE). (2) Locomotion: chair rise test, one part of the Short Physical Performance Battery Test (SPPB) was assessed. (3) Vitality: weight loss and appetite loss were recorded according to Mini-Nutritional Assessment Short Form (MNA-SF). (4) Sensory: vision and hearing loss were measured using self-reported and validated questions. (5) Psychosocial: depressive symptoms were evaluated using two questions from the Geriatric Depression Scale-15 (GDS-15). ICOPE screening tool contained nine dichotomous questions, and each question was coded as 1 or 0. We derived a summary intrinsic capacity score by adding responses to all nine dichotomous questions from five domains (possible range: 0–9). The higher the score, the better the capacity.

#### 2.5.8 Grip

Grip strength was measured using an electronic dynamometer (CAMRY, model EH10, Xiangshan, Guangdong, China). Before measurement, the dynamometer was calibrated to ensure accuracy. Participants were instructed to stand upright with feet shoulder-width apart and elbows fully extended. They were asked to grip the dynamometer with their dominant hand as firmly as possible twice, holding each grip for at least 5 s with a 30-s rest interval between measurements. If a participant was unable to stand independently, the measurement was performed in a seated position. The maximum value of the two attempts was recorded (26). According to established criteria, poor muscle strength is defined as grip strength less than 28 kg for Asian men and less than 18 kg for Asian women.

### 2.6 Ethical considerations

This study was approved by the Harbin Medical University Ethics Committee (Approval Number: HMUDQ20231116110). Prior to providing consent, all participants were fully briefed on the study’s objectives. They were informed that involvement was entirely voluntary and that they could withdraw at any point without impact on the quality of care received. Additionally, it was emphasized that all collected data would be securely stored, accessed solely by the research team, and reported in aggregate form. To protect anonymity and confidentiality, no personally identifiable information was disclosed.

### 2.7 Data analysis

SPSS 26.0, Mplus 8.3, and R 4.2.2 software was used for data analysis. Latent variable growth mixture modeling (GMM) was applied to identify trajectory categories. The model evaluation began with a single category and progressively increased the number of categories, with model fit comprehensively assessed. Key fit indices included the Akaike Information Criterion (AIC), Bayesian Information Criterion (BIC), and adjusted Bayesian Information Criterion (aBIC), where smaller values indicate a better model fit. Information entropy, ranging from 0 to 1, was used to assess classification precision, with values closer to 1 signifying more accurate classification. A statistically significant Bootstrapped Likelihood Ratio Test (BLRT) (*p* < 0.05) indicated that a model with k categories was superior to one with *k* – 1 categories. After determining the optimal number of trajectory categories, univariate analyses were performed using the chi-square test, non-parametric tests, and one-way analysis of variance (ANOVA). Variables with statistically significant differences in the univariate analyses were further included in the multivariate logistic regression analysis. To explore the relationships between influencing factors of intrinsic capacity trajectories, the “bnlearn” package in R 4.2.2 was utilized, while Bayesian network model inference was conducted using Netica software. This approach uses a graphical model (a directed acyclic graph) to visually represent variables (nodes) and the probabilistic dependencies between them (directed edges). Unlike traditional regression, a BN can illustrate both direct and indirect predictive pathways, helping to elucidate how multiple factors may interact and collectively influence the outcome (trajectory membership).

## 3 Results

### 3.1 Sample characteristics

**Figure 1:**
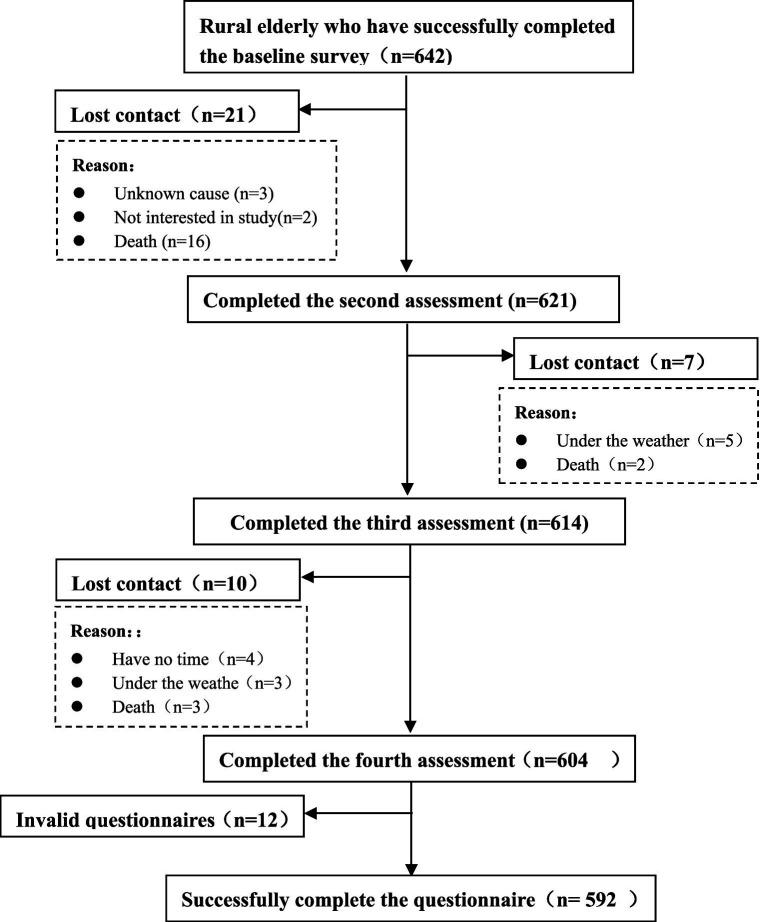
Follow-up flowchart.

In this study, a total of 642 questionnaires were distributed at baseline, and 592 valid responses were obtained after four surveys, resulting in a sample attrition rate of 7.78%, the specific follow-up process is shown in Figure 1. Among the participants, 237 (40.03%) were male, and 355 (59.97%) were female. The participants ranged in age from 60 to 86 years, with a mean age of 71.05 ± 6.20 years. Detailed demographic information is provided in Table 1.

**Table 1:** Univariate analysis of latent classes of intrinsic capacity trajectories.

Item	High functioning-stable	Medium functioning-Slow decline	Low functioning-rapid decline	*χ*^2^ /*t*	*p*-value
Age [years, (*x* ± *s*)]	68.5 ± 5.12	70.47 ± 5.94	72.59 ± 6.36	19.09	<0.001
Sex [*n*(%)]				3.67	0.16
Male	39(41.05)	106(44.17)	92(35.80)		
Female	56(58.95)	134(55.83)	165(64.20)		
Education [*n*(%)]				53.44	<0.01
Primary school and below	25(26.32)	142(59.17)	176(68.48)		
Middle school	53(55.80)	78(32.50)	69(26.85)		
High school and above	17(17.88)	20(8.33)	12(4.67)		
Marital status [*n*(%)]				27.84	<0.01
Married	83(87.37)	173(72.08)	174(67.70)		
Divorce	7(7.37)	51(21.25)	39(15.18)		
Widowed	5(5.26)	16(6.67)	44(17.12)		
Income [*n*(%)]				57.27	<0.01
Low	30(31.57)	133(55.42)	141(54.86)		
Average	32(33.68)	85(35.42)	99(38.52)		
High	33(34.74)	22(9.16)	17(6.62)		
Self-rated health [*n*(%)]				0.76	0.94
Preferably	35(36.84)	85(35.42)	89(34.63)		
Normal	44(46.32)	119(49.58)	123(47.86)		
Range	16(16.84)	36(15.00)	45(17.51)		
Number of chronic diseases [*n*(%)]				14.81	0.005
0	39(41.05)	69(28.75)	62(24.12)		
1	40(42.10)	123(51.25)	120(46.69)		
2 and above	16(16.85)	48(20.00)	75(29.19)		
Grip [*n*(%)]				13.54	0.001
Normal	70(73.68)	159(66.25)	140(54.47)		
Decline	25(26.32)	81(33.75)	117(45.53)		
Smoking [*n*(%)]				2.44	0.30
Yes	14(14.73)	35(14.58)	50(19.45)		
No	81(85.27)	205(85.42)	207(80.55)		
Drinking [*n*(%)]				2.83	0.24
Yes	22(23.16)	51(21.25)	71(27.63)		
No	73(76.84)	189(78.75)	186(72.37)		
Sleep time [*n*(%)]				1.64	0.80
<6	35(36.84)	93(38.75)	101(39.29)		
6 ~ <8	44(46.32)	118(49.17)	125(48.63)		
≥8	16(16.84)	29(12.08)	31(12.08)		
Medical insurance				0.67	0.83
Yes	93	237	256		
No	2	3	1		
Pension insurance				0.89	0.78
Yes	1	5	6		
No	94	235	251		
Loneliness (*x* ± *s*)	36.91 ± 7.61	44.99 ± 8.40	47.64 ± 11.82	40.62	<0.01
Family function (*x* ± *s*)	7.33 ± 0.92	6.13 ± 1.05	5.56 ± 1.13	95.81	<0.01
Social network (*x* ± *s*)	22.91 ± 2.81	20.47 ± 3.01	19.17 ± 3.12	53.49	<0.01
Perceived pressure (*x* ± *s*)	21.94 ± 3.11	23.83 ± 2.87	26.30 ± 3.13	85.23	<0.01
Health behavior (*x* ± *s*)	131.2 ± 30.31	106.67 ± 24.14	90.21 ± 13.30	158.91	<0.01

### 3.2 Identification and characterization of intrinsic capacity change trajectories in nursing home residents

The intrinsic capacity scores of older adults living in nursing homes at four time points were 5.27 ± 1.44, 4.89 ± 1.40, 4.66 ± 1.42, and 4.12 ± 1.50, respectively. Using these scores as observed indicators, the population was analyzed to identify potential trajectories of intrinsic capacity change through trajectory modeling, as shown in Table 2. When retaining 4 or 5 categories, the model’s AIC, BIC, and aBIC values were minimized, and the entropy value was relatively high. However, in both scenarios, the proportion of the first category was less than 10%, suggesting insufficient sample representativeness. After excluding the models with 4 and 5 categories, the model with 3 categories yielded the highest entropy value. Considering model fit indices and interpretability, the 3-category model was selected as the optimal solution.

**Table 2:** Latent class analysis model fit results for the trajectory of intrinsic capacity.

Latent class	AIC	BIC	aBIC	Entropy	Category proportion
1	5879.58	5905.88	5886.84	—	1.00
2	5803.45	5838.52	5813.12	0.79	0.81/0.19
3	5778.24	5822.08	5790.33	0.81	0.16/0.43/0.41
4	5775.29	5827.90	5789.80	0.83	0.05/0.43/0.40/0.12
5	5600.69	5662.06	5617.61	0.99	0.05/0.27/0.29/0.16/0.22

**Figure 2:**
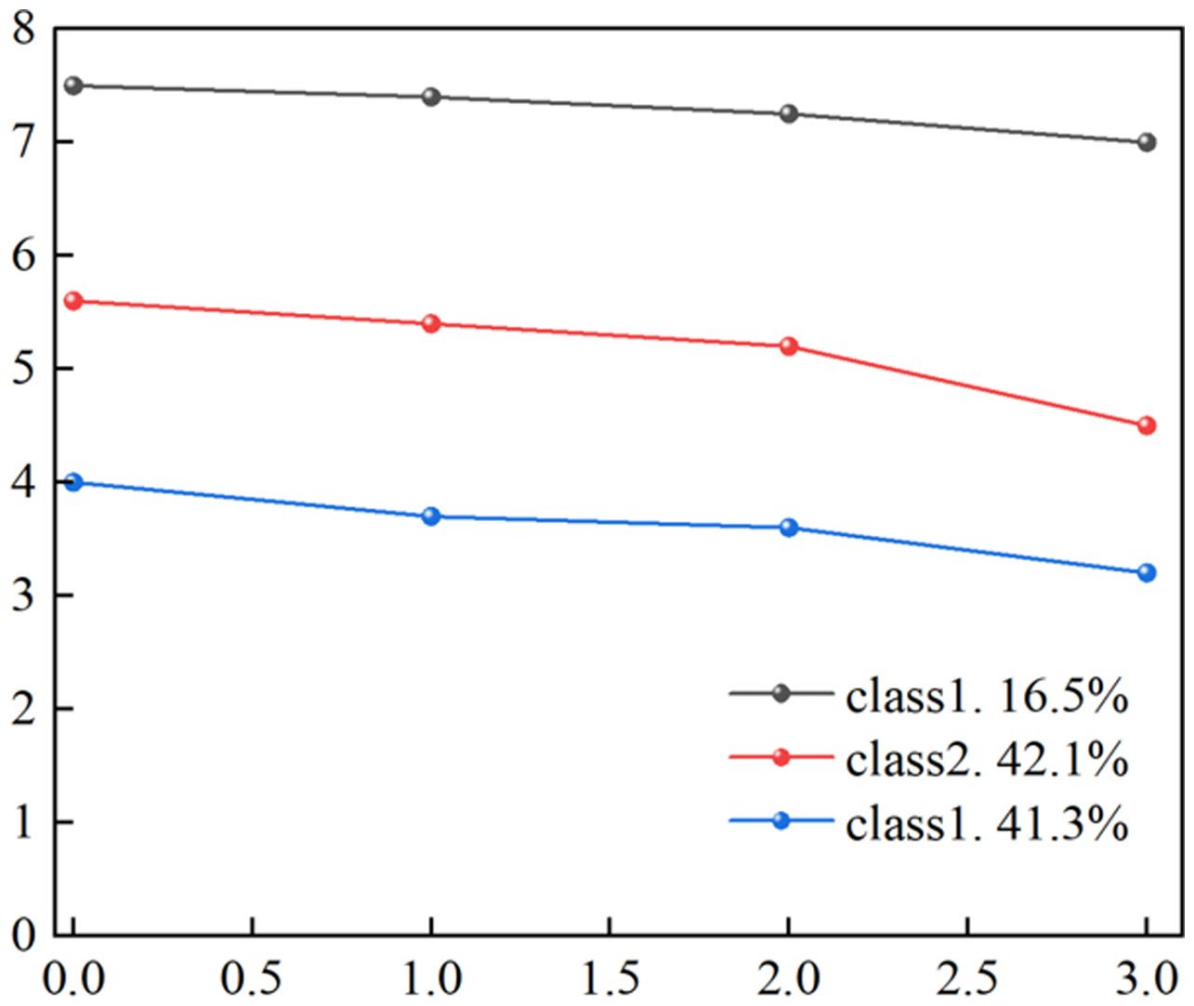
Trajectories of different latent classes of intrinsic capacity in nursing home residents.

Based on the three categories identified through GMM, the development trajectories of intrinsic capacity were established, as shown in Figure 2. Category 1: Characterized by the highest intercept and relatively stable development speed, this trajectory was classified as the “High Function-Stable Type,” comprising 95 cases (16.04%). Category 2: Defined by a moderate intercept and a slow decline in development speed, this trajectory was labeled as the ‘Medium Function-Low Decline Type,” comprising 240 cases (40.54%). Category 3: Exhibiting the lowest intercept and a rapid decline in development speed, this trajectory showed a pronounced downward trend and was categorized as the “Low Function-High Decline Type,” comprising 257 cases (43.41%).

### 3.3 Univariable analysis of intrinsic capacity trajectories in nursing home residents

The results revealed statistically significant differences in the potential intrinsic capacity development trajectories among older residents in the nursing home. These differences were associated with factors such as age, education level, marital status, economic income, number of chronic diseases, grip strength, sense of loneliness, family functioning, social network, perceived stress, and health promotion behavior scores (*p* < 0.05). Detailed results are presented in Table 1.

### 3.4 Multivariable analysis of intrinsic capacity trajectories in nursing home residents

A multinomial logistic regression analysis was conducted using the three potential categories of intrinsic capacity development trajectories among the older adults as the dependent variable, with the High Function-Stable type serving as the reference group. Variables that showed statistically significant differences in the univariate analysis were included as independent variables. Collinearity diagnostics indicated no evidence of multicollinearity (VIF: 1.012–1.144). Independent variables that were age, education level, marital status, economic income, number of chronic diseases, grip strength, sense of loneliness, family functioning, social network, perceived stress, and health promotion behavior—were included in the model, independent variables were coded as follows: education level (primary school and below = 1,0; middle school = 0,1; high school and above = 0,0); income (low = 1,0; average = 0,1; high = 0,0); marital status (unmarried/divorced = 0; married = 1); other variables were entered as their original values. The results, presented in Table 3, indicate that the number of chronic diseases, grip strength, health promotion behavior, perceived stress, family functioning, and sense of loneliness are key factors influencing the potential trajectories of intrinsic capacity development among the nursing home residents (*p* < 0.05 for all).

**Table 3:** Multivariate logistic regression analysis for the potential categories of the intrinsic capacity in nursing residents.

Variable	Low functioning-rapid decline vs. high functioning-stable				Medium functioning-slow decline vs. high functioning-stable			
	*β*	SE	*β*/SE	*P*	*β*	SE	*β*/SE	*P*
Age	−0.02	0.02	−1.16	0.24	−0.06	0.04	−1.62	0.10
Education	−0.47	0.25	−1.89	0.06	−0.62	0.39	−1.59	0.11
Income	−0.36	0.20	−1.78	0.07	−0.09	0.34	−0.26	0.79
Marital status	−0.17	0.19	−0.92	0.36	−0.01	0.34	−0.04	0.97
Number of chronic diseases	0.42	0.17	2.46	0.01	−0.95	0.33	2.92	0.00
Grip	−0.68	0.26	−2.64	0.01	−0.33	0.29	−1.39	0.22
Health behavior	0.08	0.03	2.41	0.02	0.10	0.03	3.07	0.00
Perceived pressure	−0.48	0.43	−1.11	027	−0.24	0.08	−3.15	0.00
Social network	0.07	0.04	1.68	0.09	0.07	0.08	0.86	0.39
Family function	0.51	0.12	4.30	0.00	1.58	0.22	7.32	0.00
Loneliness	−0.06	0.01	−3.88	0.00	−0.14	0.02	−5.83	0.00

### 3.5 Construction of a Bayesian network model for potential trajectories of intrinsic capacity development among the nursing home residents

**Figure 3:**
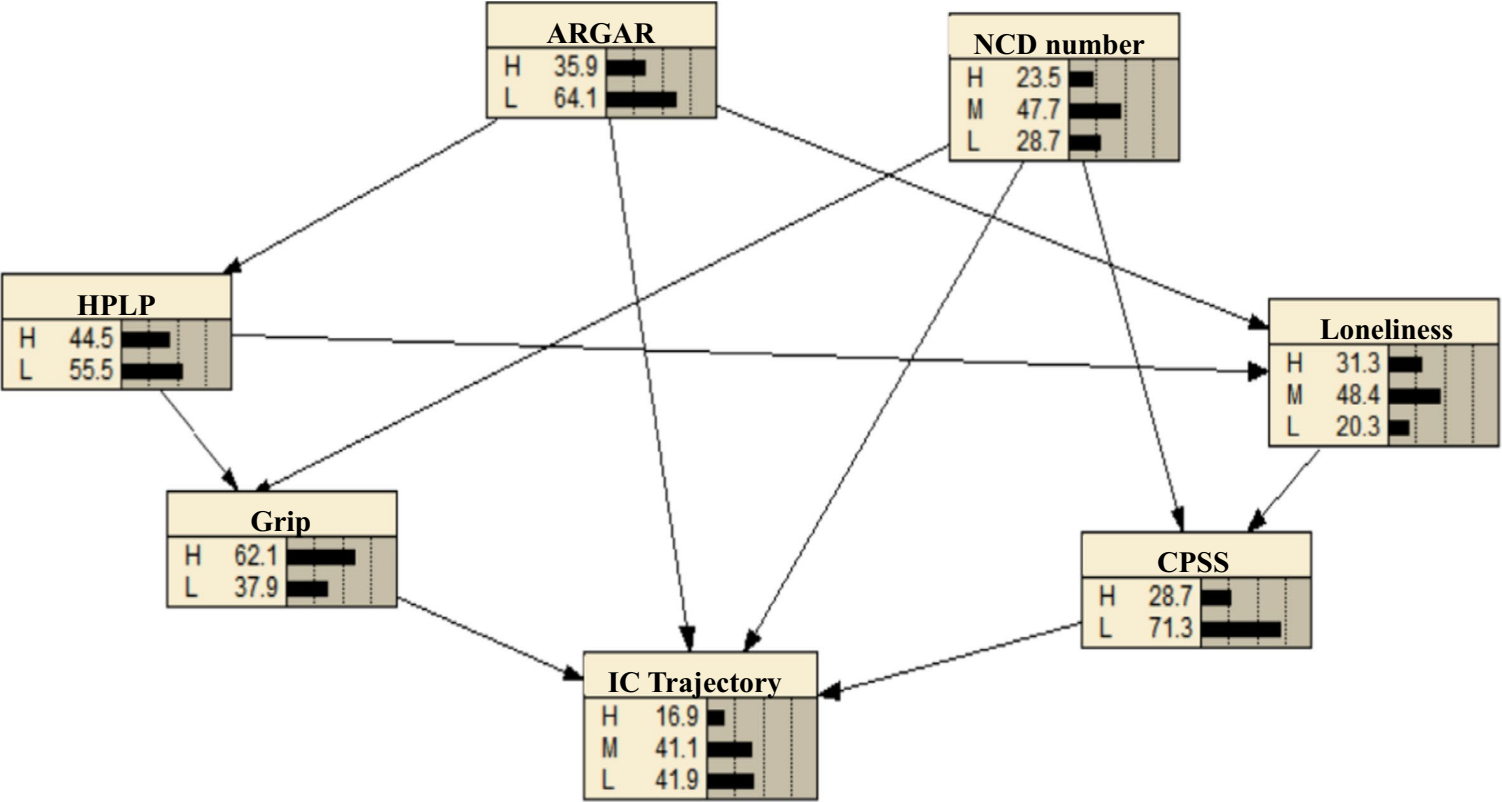
Bayesian network model of the intrinsic capacity development trajectories for nursing home residents.

Using the multivariate logistic regression analysis model, six variables with statistically significant differences were selected as network nodes to construct a Bayesian network model comprising seven nodes and 11 directed edges. The conditional probabilities for each node were calculated. The results indicate that family functioning, number of chronic diseases, grip strength, and perceived stress directly influence the potential trajectories of intrinsic capacity development among the nursing home residents. Additionally, family functioning is indirectly associated with these trajectories through health promotion behavior, grip strength, sense of loneliness, and perceived stress. Similarly, the number of chronic diseases is indirectly linked to these trajectories via grip strength and perceived stress, as illustrated in Figure 3.

### 3.6 Bayesian network model inference for latent categories of intrinsic capacity development trajectories among the nursing home residents

Table 4 presents the conditional probability table, where the latent categories of intrinsic capacity development trajectories serve as nodes. It illustrates the conditional probability dependencies between family functioning, number of chronic diseases, health behaviors, loneliness, grip strength, perceived stress, and the latent categories of intrinsic capacity development trajectories. For instance, if a study participant exhibits high family functioning, a low number of chronic diseases, high grip strength, and low perceived stress, the probability of belonging to the low-function, low-decline trajectory is 0.05.

**Table 4:** Conditional probability table of intrinsic capacity development trajectory categories among the nursing home residents.

Family function	Number of chronic diseases	Grip	Perceived pressure	Potential categories of intrinsic ability development trajectories		
				High functioning-stable	Medium functioning-slow decline	Low functioning-rapid decline
H	H	H	H	0.43	0.14	0.43
H	H	H	L	0.31	0.52	0.17
H	H	L	H	0.13	0.13	0.75
H	H	L	L	0.31	0.54	0.15
H	M	H	H	0.3	0.50	0.25
H	M	H	L	0.37	0.46	0.17
H	M	L	H	0.11	0.33	0.56
H	M	L	L	0.41	0.41	0.17
H	L	H	H	0.20	0.40	0.40
H	L	H	L	0.65	0.30	0.05
H	L	L	H	0.286	0.286	0.429
H	L	L	L	0.29	0.29	0.41
L	H	H	H	0.04	0.20	0.76
L	H	H	L	0.06	0.43	0.51
L	H	L	H	0.05	0.10	0.85
L	H	L	L	0.12	0.39	0.50
L	M	H	H	0.04	0.08	0.89
L	M	H	L	0.08	0.53	0.39
L	M	L	H	0.03	0.22	0.76
L	M	L	L	0.02	0.56	0.42
L	L	H	H	0.04	0.35	0.61
L	L	H	L	0.05	0.62	0.33
L	L	L	H	0.07	0.214	0.71
L	L	L	L	0.14	0.44	0.42

## 4 Discussion

We identified the developmental trajectories of intrinsic capacity among older adults in Chinese nursing homes and examined the determinants associated with these trajectories based on a social-ecological model. The findings highlight key modifiable determinants that are most susceptible to tailored intervention strategies.

### 4.1 Intrinsic capacity among the nursing home residents exhibits three distinct potential developmental trajectories

The development trajectory of intrinsic capacity can be classified into three potential categories: “High Functioning-Stable,” “Medium Functioning-Low Decline,” and “Low Functioning-High Decline,” indicating group heterogeneity in the intrinsic capacity trajectories among nursing home residents. The “High Functioning-Stable” group (16.5%) exhibited a higher level of intrinsic capacity at baseline, with scores remaining relatively stable throughout the follow-up period. This stability may be attributed to higher physiological and psychological health reserves, which can delay the decline in intrinsic capacity caused by aging and diseases. These findings align with those reported by Yu et al. (27), its existence provides strong empirical support for the “Successful Aging” theory, demonstrating that maintaining functional stability in advanced age is an achievable goal. The “Medium Functioning-Low Decline” group (41.3%) started with a moderate level of intrinsic capacity at baseline, followed by a gradual decline over the follow-up period. This trend highlights that the deterioration of intrinsic capacity is common among older adults as they age, consistent with other studies showing a progressive decrease in intrinsic capacity over time when measured using composite standard scores (28). The “Low Functioning-High Decline” group (42.1%) demonstrated low intrinsic capacity scores at baseline and a rapid decline thereafter. These individuals already exhibited reduced physiological reserve functions at baseline, making them more vulnerable to external factors and psychological issues. This decline may be linked to advanced age, poor physical and emotional health, and the presence of chronic diseases. Moreover, preliminary results from this study suggest that the “Low Functioning-High Decline” trajectory is a significant predictor of adverse health outcomes such as functional decline, falls, and hospitalizations. The “Low Functioning-High Decline” subgroup requires heightened clinical attention. These findings emphasize the importance of longitudinal monitoring of intrinsic capacity changes to identify heterogeneous trajectories early and implement personalized interventions to prevent further deterioration and mitigate adverse health outcomes.

### 4.2 Bayesian network model for predicting the related factors of the development trajectory of internal capacity among older adults living in nursing homes

#### 4.2.1 Direct predictive factors of intrinsic capacity development trajectories among older adults living in nursing homes: insights from a Bayesian network model

##### 4.2.1.1 Family function

The results of the Bayesian network model reveal that family functioning is a direct predictive factor for the developmental trajectory of intrinsic capacity among nursing home residents. Through risk reasoning, the model indicates that individuals with low levels of family functioning are at a higher risk of belonging to the low intrinsic capacity and high decline group. This finding provides strong support for our hypothesis that family support networks remain a critical social determinant of functional status among older adults, even within institutional care settings (29). It reveals a crucial pattern: nursing homes are not isolated enclaves. The emotional bonds, quality of interactions, and efficacy of support from the family of origin can permeate institutional barriers, continuously influencing the physical and mental health of older adults. Consequently, this significantly decelerates the decline in their intrinsic capacity and plays a pivotal role, particularly in sustaining the “Moderate Function-Low Decline” trajectory (30, 31). These observations provide a crucial complement and counterpoint to the predominant research focus on professionally led healthcare, revealing the unique value of psychosocial factors within the institutional care context. It is recommended that nursing homes transition from traditional, insular models by proactively integrating “family integration” as a core care principle. Concrete measures may include developing family empowerment programs, establishing collaborative family-staff care plans, and leveraging digital technology to facilitate remote emotional connectivity. This systematic approach transforms family function from an external variable into an actionable internal intervention resource, thereby pioneering a novel pathway for achieving integrated “medical-nursing-familial” precision health management for older adults. It should be noted that some studies have suggested that the influence of family function may diminish or be mediated by institutional care quality after relocation to a nursing home. Our findings, however, highlight its persistent direct effect, possibly because the emotional and support ties to the family of origin remain a critical psychosocial resource even within an institutional setting.

##### 4.2.1.2 The number of chronic diseases

The results of the Bayesian network model indicate that the number of chronic diseases is a direct predictive factor for the developmental trajectory of intrinsic capacity among nursing home residents. This finding provides full confirmation of the study’s initial hypothesis and substantiates the classical theory positing multimorbidity as the core pathophysiological basis driving functional decline in the older adults (32). Furthermore, the results confirm that the cumulative burden of chronic conditions, rather than any single disease, significantly accelerates both the risk and velocity of intrinsic capacity decline. This elucidates why individuals within the “Low Function-Rapid Decline” trajectory subgroup typically carry a heavier burden of disease (33). The methodological innovation of this study lies in its application of a Bayesian network model within a longitudinal trajectory analysis framework to validate this relationship. This approach not only reconfirms the association but, more critically, precisely identifies multimorbidity as a direct predictor influencing the developmental trajectory, thereby highlighting its pivotal role in the dynamic process of intrinsic capacity change. Consequently, it is imperative for the health assessment and management systems in nursing homes to transition from a “single-disease-centric” model to one that is “corely oriented around multimorbidity management.” This transition should involve the routine implementation of comprehensive geriatric assessments, utilizing the number of chronic conditions as a key indicator for risk stratification. Older adults with a high multimorbidity burden should receive early, intensive, multidisciplinary integrated interventions aimed at decelerating the rapid decline of their intrinsic capacity.

##### 4.2.1.3 Grip

The results of the Bayesian network model indicate that grip strength is a direct predictive factor for the developmental trajectory of intrinsic capacity among nursing home residents. Nursing home residents with poor grip strength are at a higher risk of belonging to the low intrinsic capacity-high decline group compared to those with normal grip strength. This finding provides robust support for the hypothesis that sarcopenia serves as a core dimension of intrinsic capacity and a key driver of overall functional decline (34), and demonstrates that upper extremity strength serves not merely as an independent indicator of physical function, but more importantly, as a barometer reflecting the overall physiological reserve and vulnerability of older adults (35, 36), its level directly predicts whether future function will tend toward stability or accelerated decline. This study provides stronger longitudinal evidence regarding its predictive value compared to research that merely reports associations or treats it as an outcome, thereby clarifying its role within the dynamic process. The findings substantiate the “muscle-function axis” theory and enrich the theoretical foundation of comprehensive geriatric assessment (37). Therefore, grip strength measurement should be implemented as a mandatory component of both admission screening and routine assessments in nursing homes to facilitate risk stratification. For older adults identified with low grip strength, comprehensive interventions—including resistance training and nutritional support (e.g., protein supplementation)—should be prioritized. By targeting muscular strength as a modifiable factor, this approach can effectively delay the decline of intrinsic capacity.

##### 4.2.1.4 Perceived stress

The results of the Bayesian network model indicate that perceived stress is a direct predictive factor for the developmental trajectory of intrinsic capacity among nursing home residents. Older adults individuals with high levels of perceived stress are at a higher risk of belonging to the low intrinsic capacity-high decline group. This finding provides strong empirical support for our research hypothesis regarding the significant role of psychological factors in the process of physical functional decline. The subjective perception of stress in older adults—transcending mere objective events—significantly accelerates the multidimensional decline of intrinsic capacity through psycho-physiological pathways. This offers a crucial psychosocial mechanism for understanding the formation of the “Low Function-Rapid Decline” trajectory (38). Prior research has predominantly focused on examining cross-sectional relationships between stress and isolated health outcomes (e.g., depression, disability) (39). This longitudinal study, employing a Bayesian network model, confirms that perceived stress serves as an independent predictor of functional trajectory patterns among institutionalized older adults. The findings underscore its role as an assessable psychological risk factor with significant implications for long-term functional prognosis, thereby addressing a critical gap in traditional biomedical models that predominantly focus on somatic indicators. For older adults with high stress perception, integrated psychological interventions, emphasizing mindfulness-based stress reduction, cognitive behavioral therapy, psychological counseling, and social support—should be systematically implemented. This approach provides both scientific rationale and a targeted entry point for achieving holistic care that integrates physical and mental health. Our finding of perceived stress as a direct predictor contrasts with some studies where its effect was fully mediated by depressive symptoms or specific health behaviors. The direct pathway identified here might reflect the unique salience of relocation stress, loss of autonomy, and environmental adjustment challenges inherent to the nursing home population, factors captured broadly by the perceived stress construct.

#### 4.2.2 Indirect predictors of intrinsic capacity development trajectories among older adults living in nursing homes: a Bayesian network model approach

##### 4.2.2.1 Healthy behavior

The results of the Bayesian network model indicate that healthy behaviors can indirectly influence the development trajectory of intrinsic capacity among older adults living in nursing homes through grip strength and feelings of loneliness. According to the World Health Organization, 60% of human health is determined by healthy behaviors. An active lifestyle (e.g., regular physical activity, balanced nutrition) enhances intrinsic capacity through dual pathways: by reinforcing functional foundations through improved physiological reserve (manifested as increased grip strength), and by fulfilling psychological needs through enhanced social interaction and self-efficacy, thereby mitigating loneliness (40). The findings further substantiate the holistic framework of the biopsychosocial model. For health behavior interventions (e.g., exercise groups, healthy meals) targeting nursing home residents, their significance extends far beyond the behaviors themselves. Caregivers should concurrently monitor and integrate social interaction elements (e.g., group exercises, communal dining), thereby simultaneously enhancing grip strength and reducing loneliness, to maximize the long-term stability of intrinsic capacity. This provides a strategic direction for designing multi-target, synergistic compound interventions.

##### 4.2.2.2 Loneliness

The results of the Bayesian network model indicate that feelings of loneliness can indirectly predict the developmental trajectory of intrinsic capacity among older adults living in nursing homes through perceived stress. According to the “Stress Amplification Hypothesis,” loneliness acts as a stressor that triggers a cascade of inflammatory responses, weakening the intrinsic capacity of the older adults and increasing their vulnerability to various psychological disorders and physical health problems (40). Nursing home residents, who often lack interaction and connection with friends and relatives, as well as adequate family and social support, face limited material and spiritual resources. These challenges, combined with physiological degeneration and psychological crises, lead to elevated levels of perceived stress. If not addressed promptly, prolonged stress can result in physiological, biochemical, and immune system changes, ultimately affecting cognitive, emotional, and physical health (41, 42), and contributing to a decline in intrinsic capacity. The findings further suggest that nursing home staff should prioritize the reduction of loneliness (e.g., by fostering social connectedness) as a key objective in institutional care. For older adults experiencing elevated loneliness, stress management techniques (e.g., mindfulness, relaxation training) should be concurrently introduced to more effectively buffer the negative impact of loneliness on functional status by interrupting this critical mediating pathway (43).

### 4.3 Limitations

This study has some limitations. First, this study collected longitudinal data at only four time points, highlighting the need for extended tracking over a longer period to comprehensively understand the long-term changes in intrinsic capacity development trajectories among older adults living in nursing homes. Additionally, further analysis is required to examine the trends and associated factors influencing longitudinal changes in intrinsic capacity. Second, the 18-month follow-up period, although aligned with recommended assessment intervals, may be insufficient to capture the full progression of decline, particularly in the “High Functioning-Stable” group. Intrinsic capacity is a multidimensional construct, and significant declines across domains might become detectable only over a longer observation window. Third, the sample for this study was exclusively drawn from Heilongjiang Province, which may limit the external generalizability of the findings. Future research should expand the scope of sampling to include multiple centers and diverse regions to enhance the generalizability of the results.

## 5 Conclusion

This study employed a longitudinal research design and identified three intrinsic capacity development trajectories among older adults living in nursing homes using a Growth Mixture Model, thereby confirming the group heterogeneity of intrinsic capacity in this population. Older adults care service personnel should focus on accurately identifying the categories of intrinsic capacity development trajectories among older adults living in nursing homes, elucidating the complex network relationships between predictive factors for each category, and developing targeted interventions based on these characteristics. Such measures can help enhance intrinsic capacity levels and prevent the onset and progression of disability in the older adults.

## Data Availability

The raw data supporting the conclusions of this article will be made available by the authors, without undue reservation.
